# The homeodomain of Eyeless regulates cell growth and antagonizes the paired domain-dependent retinal differentiation function

**DOI:** 10.1007/s13238-014-0101-9

**Published:** 2014-09-20

**Authors:** Miho Tanaka-Matakatsu, John Miller, Wei Du

**Affiliations:** Ben May Department for Cancer Research, The University of Chicago, Chicago, IL 60637 USA

**Keywords:** Pax6, eyeless, paired domain, homeodomain, cell growth, retinal differentiation

## Abstract

Pax6 and its *Drosophila* homolog Eyeless (Ey) play essential roles during eye development. Ey/Pax6 contains two distinct DNA binding domains, a Paired domain (PD) and a Homeodomain (HD). While Ey/Pax6 PD is required for the expression of key regulators of retinal development, relatively little is known about the HD-dependent Ey function. In this study, we used the UAS/GAL4 system to determine the functions of different Ey domains on cell growth and on retinal development. We showed that Ey can promote cell growth, which requires the HD but not the PD. In contrast, the ability of Ey to activate Ato expression and induce ectopic eye formation requires the PD but not the HD. Interestingly, deletion of the HD enhanced Ey-dependent ectopic eye induction while overexpression of the HD only Ey forms antagonizes ectopic eye induction. These studies revealed a novel function of Ey HD on cell growth and a novel antagonistic effect of Ey HD on Ey PD-dependent eye induction. We further show the third helix of the Ey HD can directly interact with the RED subdomain in Ey PD and that deletion of the HD increased the binding of Ey PD to its target. These results suggest that the direct interaction between the HD and the PD potentially mediates their antagonistic effects. Since different Ey splicing forms are expressed in overlapping regions during normal development, we speculate that the expression ratios of the different Ey splice forms potentially contribute to the regulation of growth and differentiation of these tissues.

**Electronic supplementary material:**

The online version of this article (doi:10.1007/s13238-014-0101-9) contains supplementary material, which is available to authorized users.

## INTRODUCTION

Pax6 is an evolutionary conserved transcription factor and is indispensable for eye development. Mutations in Pax6 genes cause eye developmental defects in a wide range of species. In *Drosophila* and mouse, null mutations of Pax6 orthologs *eyeless* (*ey*) and *small eye* (*Sey*) cause severe defects in the eye (Hill et al., 1991; Quiring et al., 1994). In humans, heterozygous Pax6 gene mutations are associated with eye disorders such as aniridia, Peters anomaly, and keratitis (Glaser et al., 1992; Jordan et al., 1992; Mirzayans et al., 1995; van Heyningen and Williamson, 2002). The Pax6 proteins contain two highly conserved DNA binding domains, a paired domain (PD) and a homeodomain (HD), and a transactivation domain in the C-termini. Interestingly, many Pax6 mutations that cause eye developmental defects have mutations that disrupt the PD (van Heyningen and Williamson, 2002). In addition, the PD but not the HD of Ey is shown to be required for ectopic eye induction in *Drosophila* (Punzo et al., 2001; Punzo et al., 2002). The PD is composed of a bipartite DNA binding domain that consists of two helix-turn-helix motifs, the PAI and the RED subdomains. Individual PAI and RED subdomains make contact to the known consensus Pax6 DNA binding sequences: WWNMCRMNTSANTGRRY and both PAI and RED subdomains contribute to the overall binding of the PD (Czerny et al., 1993; Epstein et al., 1994a, 1994b; Treisman et al., 1991; Xu et al., 1999; Xu et al., 1995). PD binding sites have been identified in the enhancer regions of the retinal determination factors (RD factors) including Sine Oculis (So), Eyes absent (Eya), Optix, and in the 3′ eye enhancer of the proneural gene Atonal. Targeted disruptions of the PD binding sites have been shown to block activation of these target genes by Ey (Ostrin et al., 2006; Tanaka-Matakatsu and Du, 2008; Tanaka-Matakatsu et al., 2014; Xu et al., 1999; Zhang et al., 2006). Therefore PD-dependent transcription activation by Ey plays critical roles in the activation of these target genes during normal eye development.

In contrast to the PD-dependent Ey function, much less is known about the HD-dependent function of Ey. The homeodomain of Ey contains a stretch of 60 amino acids that is conserved in the large family of the Homeobox containing transcription factors. The 60-amino-acid-long HD consists of three alpha-helical structures, α1, α2 and α3. The α3 is the DNA recognition helical structure that directly binds to the TAAT sequences locates in the DNA major groove. The HD of Pax6 preferentially binds as a dimer to the palindromic DNA binding site: TAATYNRATTA (Y is C or T; R is A or G; N is any nucleotide), which is known as the P3 site. It was reported that the P3 sites play important roles in the activation of rhodopsin family genes by the HD-dependent Pax6 function (Mismer and Rubin, 1989; Papatsenko et al., 2001; Sheng et al., 1997). Interestingly, a conserved alternative splicing form of Ey/Pax6 that contains the HD but lacks the PD has been reported in a wide range of species (Carriere et al., 1993; Epstein et al., 1994a; Jaworski et al., 1997; Mishra et al., 2002; Papatsenko et al., 2001; Sheng et al., 1997). These observations suggest that the HD-form of Ey/Pax6 also plays important roles in development.

Although early studies of Ey/Pax focus on their critical role in eye development due to their striking ability to induce eye in *Drosophila* and frog *Xenopus laevis* upon misexpression (Chow et al., 1999; Gehring, 1996; Halder et al., 1995; Nornes et al., 1998), Ey/Pax6 has other functions in addition to eye development. This is consistent with the observation that human Pax6 homozygote mutations display defects in non-eye regions such as nose, forebrain, hindbrain and pancreas (Hill et al., 1991; St-Onge et al., 1997). Moreover Pax6 has been shown to have oncogenic potential and regulate cell growth in cultured human cells and mouse model system (Li and Eccles, 2012; Mascarenhas et al., 2009; Robson et al., 2006; Walcher et al., 2013). Interestingly, Pax6(5a), a Pax6 splicing variant that removes the PAI subdomain of PD, and its *Drosophila* functional and structural homologue Eyegone (Eyg) have been shown to regulate cell growth and proliferation (Dominguez et al., 2004; Jang et al., 2003). It is likely that Eyg/Pax5(5a) use RED and HD to bind their targets. Therefore, whether Pax6 will promote growth or differentiation will be regulated by the relative levels of distinct Pax6 splicing forms in the cell. Indeed, several lines of evidence revealed that many Pax6 target genes are regulated in a context specific manner (Kiselev et al., 2012; Wolf et al., 2009; Xie et al., 2013).

In this study, we characterized the roles of the HD-dependent Ey function and show that the HD and C-terminal transactivation domain of Ey promotes cell growth. In addition, we show that the Ey-HD physically interacts with the RED subdomain of the PD, and this interaction interferes with the PD-dependent transcriptional activation of the Ato 3′ eye disc enhancer. These results provide new insights into the roles of the different Ey splice forms on retinal cell fate determination and cell growth during development.

## RESULTS

### Eyeless promotes context-dependent cell growth in wing discs

**Figure 1:**
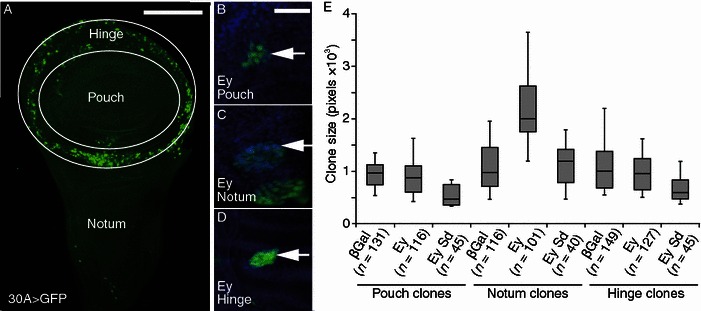
**Eyeless promotes context-dependent cell growth in wing discs**. (A) Diagram of third instar larval wing disc shows three distinct regions. The region between outer- and inner-white circles will develop into the fly wing hinge, while inner pouch region forms fly wing blade. The bottom Notum region will develop into the future scutellum. (B–D) Ey expressing Flp-out clones (marked with GFP in green) in the three distinct regions (white arrows). Wing discs were counter stained with DAPI (blue). The median size clones are shown. The scale bars are 50 μm in (A) and 100 μm in (B–D). (E) Box-whisker plot for FLP-out ectopic clone size induced in the pouch, notum and hinge regions. Number of clones (*n*) are indicated in the figure for each genotype. Single heat shock at 34°C for 10 min was applied at AEL 47 ± 1 h larva and cultured additional 48 h at 25°C. The *P* values to the β-Gal expressing control clone are: *P* = 0.064 (Ey), *P* = 0.58 × 10^−9^ (Ey Sd) in pouch. The *P* = 5.62 × 10^−5^ (Ey) and *P* = 0.32 (Ey Sd) in notum, the *P* < 0.95 (Ey), *P* = 2.28 × 10^−7^ (Ey Sd) in hinge

Recent studies indicate that Pax6 regulates multiple transcriptional networks that regulate cell proliferation as well as differentiation (Farhy et al., 2013). To characterize the effect of Eyeless on the rate of cell growth and proliferation *in vivo*, we used the “flip-out” GAL4 driver (Act>GAL4) approach to co-activate permanent, heritable expression of UAS regulated targets in random clones of cells (Duman-Scheel et al., 2002; Neufeld et al., 1998; Xin et al., 2002). This technique uses heat shock to induce FLP recombinase, which induces the generation of random clones of GAL4 expression cells at precisely defined time point for cell growth analysis. Specifically 47 ± 1 h larvae were heat shocked to turn on Gal4 expression, which activates UAS-target gene expression including the UAS-GFP in discrete clones in imaginal discs. After another 48 h of growth, the wing discs were dissected and the size of the clones was analyzed. Wing disc can be divided into the wing pouch, hinge, and the notum region (Fig. 1A). Analysis of the control and the Ey-expressing clones in different wing disc regions revealed that the ability of Ey to promote growth varies depending on the region in which the clone is located. While Ey expressing clones are significantly larger than the β-Gal controls located in the notum region, no significant difference were observed for the clones located in the pouch or hinge regions (Fig. 1B–E). These results show that Ey can promote cell growth in a context-dependent manner.

The context specificity of Ey-dependent growth suggests that there are different signaling pathways in the different disc regions that modulate the effect of Ey overexpression on tissue growth. Scalloped (Sd), a component of the Hippo signaling pathway, is highly expressed in the pouch of the wing disc and very weakly expressed in the notum region (Campbell et al., 1992). To determine whether Sd may contribute to the context-dependent growth of Ey in wing disc, we examined the effect of expressing Sd with Ey. Coexpression of Sd with Ey significantly inhibited the ability of Ey to promote cell growth in the notum region (Fig. 1E). In addition, coexpression of Sd with Ey can also inhibit cell growth in the pouch and hinge regions as shown by decreased clone size (Fig. 1E). Therefore Sd can antagonize the ability of Ey to promote cell growth and the high level of Sd in the wing pouch region potentially contributes to the inability of Ey to promote growth in the pouch.

### The Ey homeodomain and C-terminal region, but not the paired domain, are required for Ey-dependent growth

**Figure 2:**
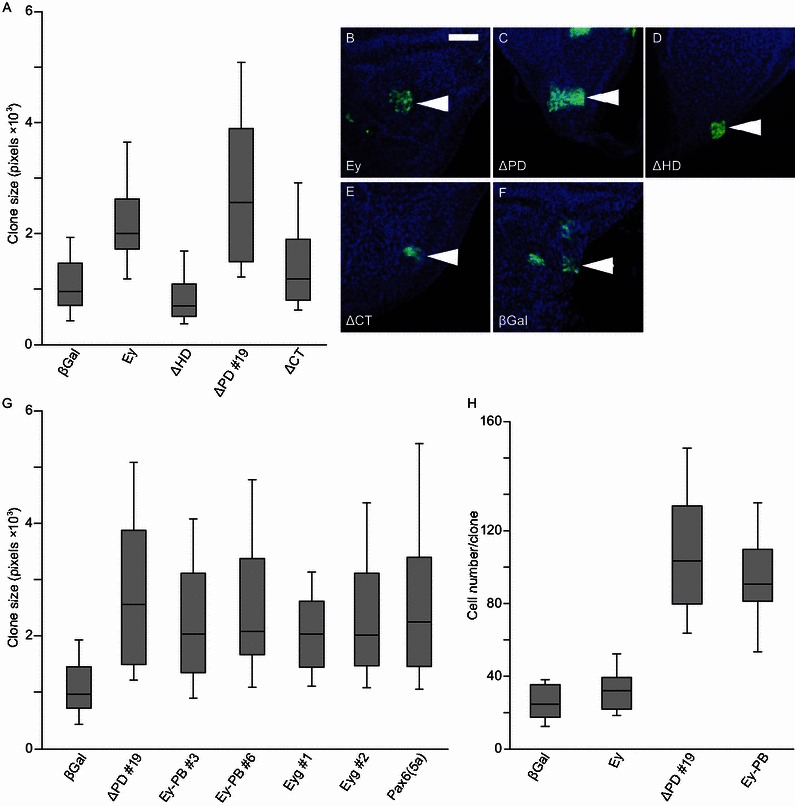
**The HD and C-terminal transactivation domain are required for the ability of Ey to promote cell growth and proliferation**. (A and G) Box-whisker plot of ectopic clone size induced in the notum region of the third instar wing disc. Minimum 50 clones and maximum 150 clone sizes were analyzed in this experiment. A single heat shock at 34°C for 10 min was applied at AEL 47 ± 1 h larva and cultured for an additional 48 h at 25°C. (A) The effect of expressing WT Ey or different Ey domain deletion constructs on clone sizes. The sizes of WT Ey and the PD deletion Ey expressing clones were significantly larger than those of the β-Gal control clones. The *P* values are: *P* = 5.62 × 10^−5^ (Ey and β-Gal control), *P* = 3.12 × 10^−15^ (∆PD and β-Gal control), and *P* = 4.78 × 10^−6^ (∆PD and Ey). On the other hand, the sizes of the HD deletion (∆HD) or the C-terminal transactivation domain deletion (∆CT) clones were similar to that of to the β-Gal expressing control clones. (B–F) FLP-out clones in the wing notum region. Median values of clones are shown from A (white arrowheads). Genotypes are indicated in each bottom panel. Clones expressing truncated Ey were labelled with GFP (green). Wing discs were counter stained with DAPI (blue). The scale is 50 μm. (G) Constructs contain HD and C-terminal transactivation domains increased clone size. *P* values to the β-Gal control clones are *P* < 1.0 × 10^−5^. (H) Statistical analysis of cell numbers in each genotyped clones (*n* = 20). *P* values to the β-Gal expressing control are: *P* = 0.037 (Ey), *P* < 8.12 × 10^−14^ (∆PD, Ey-PB)

We further characterized the effect of Ey-dependent growth through the expression of Ey deletion constructs that lack the PD (Ey ∆PD), HD (Ey ∆HD), or the C-terminal transactivation domain (Ey ∆CT). We focused our analysis to the notum region since WT Ey showed significant growth effects in this region (Fig. 2A, 2B and 2F). Interestingly, the sizes of the Ey ∆HD and the Ey ∆CT clones were significantly smaller than that of the WT Ey clones and similar to that of the β-Gal control clones (Fig. 2A, 2B, 2D, 2E and 2F). These results indicate that both the HD and the C-terminal transactivation domain are required for the ability of Ey to promote growth. On the other hand, the size of the Ey ∆PD clones was actually slightly larger than that of the WT Ey clones and both Ey ∆PD and WT Ey were significantly larger than that of the β-Gal control clones (Fig. 2A, 2C and 2F). Taken together, these results suggest that the ability of Ey to promote cell growth in the wing notum depends on its HD DNA binding domain and the transcription activation domain. On the other hand, the Ey PD DNA binding domain is not required for the growth promotion effects and may actually antagonize the Ey HD-mediated growth effect.

### The HD-only form of Ey (Ey-PB), Eyg, and Pax6(5a) show similar growth promoting activities as Ey ∆PD in developing notum region

To further demonstrate that the observed effect of Ey on cell growth is mediated by the HD and to characterize the growth effect of Ey-PB, the HD-only splicing variant of Ey (Fig. S1), we generated UAS-Ey-PB transgenic flies. As shown in Fig. 2, expression of Ey-PB also significantly increased clone sizes, similar to that of Ey ∆PD (Fig. 2G). Eyg and its human homologue Pax6(5a) were shown previously to regulate cell growth and proliferation in the developing eye (Dominguez et al., 2004; Jang et al., 2003; Yao and Sun, 2005). We compared the effects of Eyg and Pax6(5a) on cell growth in our assay system. Clones expressing Eyg or Pax6(5a) were significantly larger than those expressing the β-Gal control but were similar to those expressing Ey ∆PD or the HD-only splicing variant Ey-PB (Fig. 2G). Therefore, the HD-only splicing variant Ey-PB, Ey ∆PD, Eyg and Pax6(5a) show similar growth promotion effects.

To determine whether Ey-induced growth is mediated by increased cell proliferation, we determined whether expression of the Ey constructs also increased cell numbers. As shown in Fig. 2H, both Ey-PB and Ey ∆PD increased cell numbers, suggesting that Ey promotes both cell proliferation and cell growth to induce larger clone sizes.

### The Ey HD antagonizes Ey PD-dependent function on retinal determination *in vivo*

**Figure 3:**
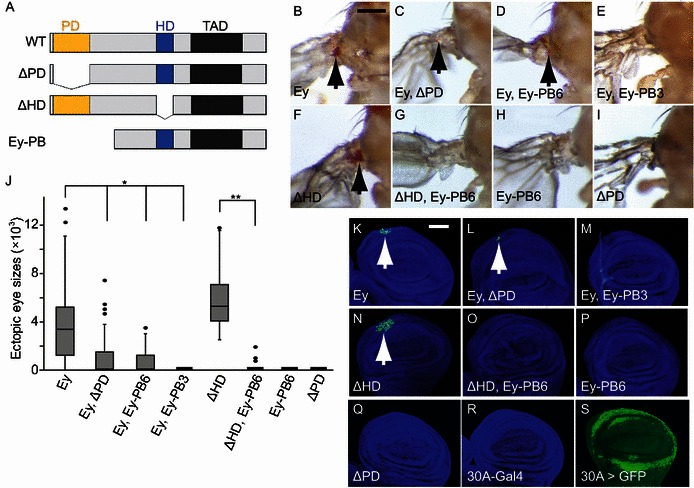
**Ey Homeodomain inhibits Ey PD-dependent 3.6BB-GFP activation**. (A) Diagram of Ey constructs. The PD (filled yellow box) and HD (filled blue box) are DNA binding domains, TAD (filled gray box) is the C-terminal transactivation domain. (B–E) Co-expression of Ey constructs that contain only HD (∆PD or Ey-PB) inhibited WT Ey to induce ectopic eye on the hinge. (F and G) Co-expression of Ey-PB inhibits ∆HD induced ectopic eye. Genotypes were listed at the bottom of the panel. Ey-PB6 and Ey-PB3 are HD type Ey isoforms inserted in different chromosomal region. Ey-PB (H) and ∆PD (I) were unable to induce ectopic eye. (J) Box-whisker plot of ectopic eye sized induced on the hinge. *n* = 50 for each genotype. Each *P* value to Ey is *P* < 1.59 × 10^−7^ (*) and to ∆HD is *P* = 1.0 × 10^−29^ (**). (K–R) Ectopic expression of the Ato-3.6BB-GFP was revealed in the wing disc under the 30A-gal4 driver. Ey constructs that contain only HD inhibited WT Ey (K–M, white arrows) or Ey ∆HD (N–O, white arrow). Ectopic expression of either Ey-PB (P) or ∆PD (Q) was unable to activate the Ato-3.6BB-GFP. In the third instar wing disc the Ato-3.6BB-GFP had no expression (R). The 30A-gal4 drives expression in the imaginal hinge region (S). Genotypes listed at the bottom of the panel. Median value wing disc images were shown. Discs were counterstained with DAPI (blue). The scale bars for wing discs and adult hinge are 50 μm and 200 μm, respectively

Ey PD-dependent activity has been shown to induce retinal determination and activate the proneural gene Ato 3′ enhancer (Tanaka-Matakatsu and Du, 2008; Tanaka-Matakatsu et al., 2014; Zhang et al., 2006). Ectopic expression of Ey using the 30A-Gal4 driver (Brand and Perrimon, 1993), which drives expression in a ring like hinge domain surrounding the wing pouch (Fig. 3S), was able to activate Ato 3.6BB-GFP in a subset of cells near the AP boundary (Fig. 3K) and induce ectopic eye formation in the hinge region (Fig. 3B). Interestingly, expression of the HD deletion Ey construct (Ey ΔHD) induced increased Ato 3.6BB-GFP expression (Fig. 3K and 3N) and larger ectopic eye sizes compared to those induced by WT Ey (Fig. 3B, 3F and 3J). In contrast, expression of Ey containing the PD deletion (Ey ΔPD) was unable to activate Ato 3.6BB-GFP or induce ectopic eyes in adult wing hinge (Figs. 3I and 3Q). Similarly, expression of Ey-PB, the alternative splice form of Ey that only contains the HD (Fig. S1), is unable to induce ectopic Ato 3′ enhancer activation or ectopic eye formation in the wing hinge region (Fig. 3H and 3P). Since the expression levels of these deletion constructs are not significantly different (Fig. S2) and since Ey ΔPD can promote cell growth (Fig. 2) while Ey ΔHD can promote retinal differentiation (Fig. 3F and 3N), our results show that PD is required for Ey activation of the Ato 3′ enhancer and suggest that the HD may have an inhibitory effect on Ey PD to activate the Ato 3′ enhancer.

To further characterize the *in vivo* functional interactions between Ey PD and Ey HD, we tested the effect of expressing WT Ey with the Ey ΔPD or Ey-PB. Interestingly, expression of either ΔPD or Ey-PB significantly decreased the ability of WT Ey to activate Ato 3′ enhancer expression and to induce ectopic eye formation (Fig. 3C, 3D, 3E, 3J, 3L and 3M, *P* < 0.0001). Furthermore, while Ey ΔHD can induce more Ato 3′ enhancer activation and larger ectopic eye formation in the wing hinge region (Fig. 3F, 3J and 3N), coexpression of Ey-PB significantly inhibited both ectopic Ato enhancer activation and ectopic eye formation (Fig. 3G, 3J and 3O). Therefore, the Ey HD can inhibit the ectopic eye induction function of the Ey PD even if the two domains are present in two different proteins.

### Ey HD interacts with Ey PD through the RED subdomain

**Figure 4:**
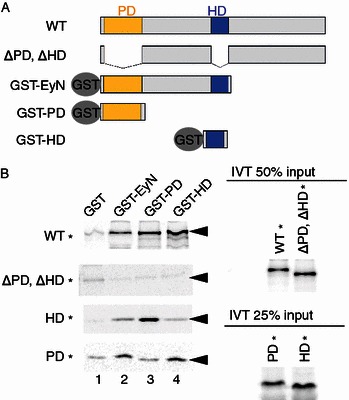
**Ey PD and HD physically interact with each other**. (A) Diagram of Ey WT and deletion constructs and GST-fusion Ey proteins. PD and HD are shown in yellow and blue filled boxes, respectively. (B) GST-pull down experiments. ^35^S-labelled *in vitro* translated proteins were marked with *. A 50% or 25% input of the ^35^S labeled proteins was shown on the right panel. ^35^S-labeled WT Ey binds and pulled down any GST-fusion Ey deletion proteins. Deletion of both PD and HD (∆PD, ∆HD*) abolished interaction and was not pulled down by any GST-fusion proteins. HD* or PD* was pulled down by GST-PD or GST-HD reciprocally. Heterologous domain interaction increased pulled down

Several previous studies have suggested that the direct protein-protein interactions between HD and PD mediate the functional antagonisms between the HD containing selector proteins, such as Proboscipedia (PB) and Antenapedia (Ant), and the PD-dependent Ey (Benassayag et al., 2003; Plaza et al., 2001). Therefore we hypothesize that the observed *in vivo* functional interactions between Ey HD and Ey PD are mediated by their direct protein-protein interactions between the two domains. GST pull down assays were carried out to directly test this possibility. Incubation of ^35^S-Met labeled WT Ey with immobilized glutathione S-transferase (GST) or GST tagged Ey deletion proteins EyN, PD and HD (Fig. 4A) revealed that WT Ey (WT*) was bound specifically to all three of the GST-Ey deletion proteins (Fig. 4B, WT*). On the other hand, ^35^S-Met labeled Ey with deletions of both the PD and HD failed to interact with any of the GST Ey deletion constructs (Fig. 4B, ΔPD, ΔHD*). These observations suggest that the PD and HD of Ey are critical for the observed interactions. Interestingly, ^35^S-Met labeled Ey paired domain alone (PD*) showed preferential interaction with the GST constructs that contains HD (Fig. 4B, GST-EyN and GST-HD). Conversely, ^35^S-Met labeled Ey homeodomain (HD*) showed strong interaction with the GST-PD (Fig. 4B). Therefore, the Ey HD and PD can directly interact with each other.

**Figure 5:**
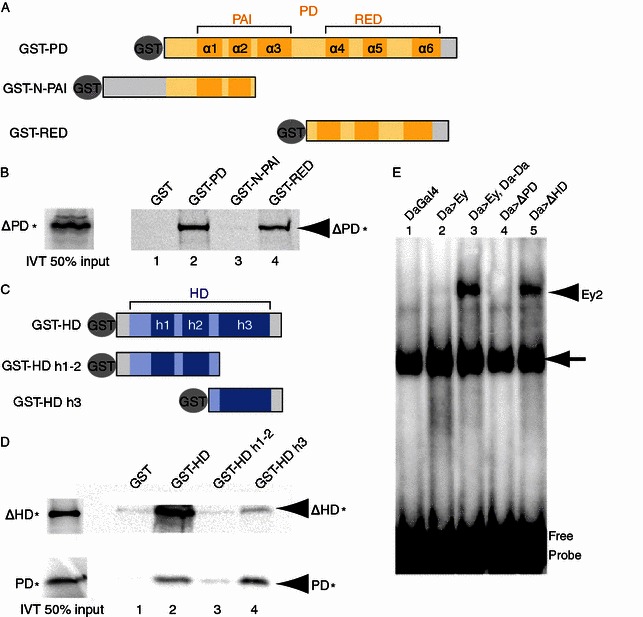
**RED subdomain of PD physically interacts with the third helix of HD**. (A) Diagram of PD deletion constructs. The PD consists of the N-terminal PAI subdomain, C-terminal RED subdomain, and a linker. Both PAI and RED contain three alpha helical structures (α1 to α6, in dark yellow). (B) ^35^S-labelled ∆PD* (which contains HD) was pulled down by GST-fusion proteins containing the RED subdomain (lanes 2 and 4) but not by the GST fusion with the N-terminal PAI domain. (C) Diagram of HD deletion constructs. The HD contains three alpha helical structures (indicated h1 to h3, in dark blue), and the 3rd helix is used for direct DNA contact. (D) ^35^S-labelled ∆HD and PD proteins (which contain PD) were pulled down by GST-fusion proteins contains h3 helix (lanes 2 and 4) but not by GST fusion with the first and second helixes of HD (lane 3). (E) Electrophoretic mobility shift assay (EMSA) using Ey2 sequence from the Ato 3.6BB eye enhancer as probes. Embryonic extracts with indicated Da-Gal4 driven UAS-target gene expression were used in EMSA using ^32^P-labeled Ey2 probe. Arrowhead indicates specific band shift in Ey, Da-Da and ∆HD genotypes. Arrow indicates non-specific band shift. Specific band shift showed significant difference between Da>Ey (lane 2) and Da>∆HD (lane 5) while nonspecific bindings were similar (arrow, lanes 2, 3 and 5)

Crystal structure studies showed that the PD consists of an N-terminal PAI subdomain, a C-terminal RED subdomain, and a linker between the two (Fig. 5A) (Jun and Desplan, 1996; Xu et al., 1999) while the HD consists of three α-helices (Fig. 5C) (Gehring et al., 1994). We generated additional GST-PD deletion constructs (Fig. 5A) to further define the subdomains of PD that interact with Ey HD. As shown in Fig. 5, ^35^S-Met labeled Ey ΔPD, which contains the Ey HD, was retained specifically by the PD through the RED subdomain (Fig. 5B). Therefore the RED subdomain of PD mediates the interaction between Ey PD and HD. We further determined sequences within HD that interacted with Ey PD, we found that GST-fusion proteins that contain the third helix of the HD (GST-HD h3) were able to interact with the PD (Fig. 5D, lanes 2 and 4). On the other hand, GST-fusion protein that contains the first and second helixes (GST-HD h1–2) was unable to bind the PD (Fig. 5D, lane 3). Taken together, our results show that the RED subdomain of Ey PD interacts with the helix 3 of Ey HD.

### The Ey HD inhibits Ey-PD dependent binding to the Ey binding site in the Ato 3′ enhancer

The interactions between the Ey-PD and Ey-HD can potentially alter the DNA binding of these DNA binding domains. We carried out EMSA to test if HD removal affects Ey PD to bind its target site. The Ato 3′ enhancer is an established direct target of Ey that is mediated by Ey-PD. We showed previously that Da homodimer directly interacts with Ey and promotes Ey binding to the Ey binding site in the Ato 3′ enhancer (Tanaka-Matakatsu et al., 2014). Consistent with this, while very weak Ey binding activity to the Ey2 site was detected in embryonic extracts with WT Ey expression alone (Fig. 5E, lane 2, arrowhead), expression of WT Ey with Da-Da linked dimer extracts significantly increased Ey binding to the Ey2 (Fig. 5E, lane 3). Interestingly, expression of Ey ∆HD, which has deletion of the HD domain, significantly increased binding of Ey to Ey2 binding site while expression of Ey ∆PD, which has deletion of the PD, did not. These results are consistent with the idea that interactions between the PD and the HD alters the DNA binding activities of PD, which potentially contributes to the observed inhibitory effects of HD on Ey PD to induce Ato expression and ectopic eye formation.

## DISCUSSION

In this manuscript, we showed that the HD-only form of Ey can promote cell growth and proliferation during imaginal disc development. In addition, we showed that the HD and PD of Ey can directly interact with each other and that the HD-only form of Ey can antagonize the function of Ey PD-dependent retinal differentiation function. These results suggest that the functions of Ey are potentially regulated by factors that promote or inhibit the interactions between the two domains. In addition, the relative abundance of the HD and PD forms of Ey may contribute to the coordinated control of cell growth/proliferation and differentiation by Ey.

### Ey PD-HD interaction negatively regulates Ato activation in eye development

Ato is a transcription factor required for the induction of photoreceptor differentiation (Jarman and Groves, 2013; Jarman et al., 1995). The Ato 3′ enhancer, which controls the initial Ato expression in the developing third instar eye disc, is regulated by Ey/Pax6, bHLH protein Daughterless dimer, Sine oculis and Eya (Tanaka-Matakatsu and Du, 2008; Tanaka-Matakatsu et al. 2014; Zhang et al., 2006). Results presented here reveal an interesting regulatory mechanism for the retinal determination function of Ey: the intramolecular interaction between the RED subdomain of the PD and the HD leads to an inhibition of the PD-dependent retinal determination function. Interestingly, expression of the Pax6 isoform that lacks the PD causes a microphthalmic phenotype in Pax6(+/+) mice, suggesting that the mammalian Pax6 isoform that contains only the HD antagonizes the WT Pax6 function (Kim and Lauderdale, 2006), similar to our observed effects of expressing Ey HD only isoforms in flies (Fig. 3). Furthermore, overexpression or ectopic expression of various homeobox-containing proteins has been shown to inhibit eye development (Chadwick et al., 1990; Gibson et al., 1990; Jiao et al., 2001; Yao et al., 1999). It is possible that the interactions between these different HD with Ey PD may contribute to the observed inhibition of eye development. Indeed, HD-containing protein Engrailed interacts with Pax6 through the PD (Plaza et al., 1997). On the other hand, the protein-protein interaction between HoxB1 and Pax6 was shown to increase the Pax6 transcriptional activity using an artificial reporter with six consensus Pax6 PD-binding sites in Hela cells (Mikkola et al., 2001). It is possible that different Pax6 targets are differentially affected by the HD and PD interaction due to the presence of additional context dependent partners.

Our previous studies showed that Da homodimer interacted with the RED subdomain of Ey and promoted Ey binding to the Ey2 binding site in the Ato 3′ enhancer (Tanaka-Matakatsu et al., 2014). While binding of the Da dimer to the E-box site within the composite Ey2 binding site is important, it is possible that binding of the Da dimer with the Ey PD may interfere the Ey PD interaction with the Ey HD and thus contribute to the increased Ey PD-dependent function.

### Ey homeodomain can regulate cell proliferation and growth in imaginal disc development

Eye development requires not only the correct specifications of cell types, but also the control of their size and cell number. Coupling between cell-cycle exit and onset of differentiation is a common feature throughout development. In the third instar fly eye, photoreceptor differentiation is initiated at the posterior margin of the eye disc, forming a typical groove-like structure called the morphogenetic furrow (MF). The retinal progenitor cells immediately ahead of the MF adopted a pre-proneural (PPN) state just poised prior to the neuronal differentiation (Bessa et al., 2002; Greenwood and Struhl, 1999; Silver and Rebay, 2005). Ey together with retinal cell fate determination factors directly regulate Ato, which promotes photoreceptor differentiation and regulates the expression of the cdk inhibitor Dacapo (Sukhanova et al., 2007). Although Pax6 has been implicated in both proliferation and differentiation of invertebrate development, whether fly homolog Ey in these processes are largely unknown. Instead Eyg has been reported to regulate growth at the eye midline organizer in response to Notch signaling (Chao et al., 2004; Dominguez et al., 2004). Eyg is related to a splicing isoform of vertebrate Pax6(5a), which contains an extra exon that disrupts the N-terminal PAI subdomain of the PD. Therefore Pax6(5a) binds to distinct DNA target sites through the C-terminal RED subdomain and perhaps the HD (Epstein et al., 1994b; Kozmik et al., 1997). As expected, Eyg and Pax6(5a) are unable to induce ectopic eye formation in the wing primordium but can drive significant wing disc overgrowth when expressed using the DPP-Gal4 driver. Interestingly, using our clonal analysis to determine the direct role of these genes on cell growth and proliferation, we showed that Eyg and Pax6(5a) can promote cell growth similar to the Ey ∆PD and the Ey-PB splicing form. Although little is known of the function of the Ey-PB form, we report here that Ey-PB is expressed similarly as the full length Ey during eye development. Since full length Ey and Ey-PB have distinct ability to regulate cell differentiation and cell growth and proliferation, it is interesting to speculate that the relative expression of the two proteins can potentially regulate cell proliferation and retinal differentiation in developing tissues.

## MATERIALS AND METHODS

### Fly strains, misexpression and mosaic clone analysis

Drosophila culture was performed at 25°C on standard cornmeal-yeast medium. For wing disc clone analysis, embryos were collected every 2 h, then a heat shock was applied for 10 min at 34°C at AEL 47 ± 1 h. Larvae were cultured for an additional 48 h at 25°C before dissection. Fly strains used in this study were shown here. Multiple lines were tested to verify that we got consistent results. For some strains, we re-hopped and generated new insertions on different chromosome. UAS-ey (BL6294, on the 2^nd^ chromosome), UAS-ey (rehopped on the 3^rd^ chromosome from the BL6294), UAS-∆PD #8-6 and #19 (Weasner et al., 2009), UAS-∆HD (Weasner et al., 2009) (Punzo et al., 2001) and tested at least 3 lines that rehopped onto the 2^nd^ choromosome from the 3^rd^ chromosome), UAS-∆CT (Clements et al., 2009), UAS-eyg #1 (Yao and Sun, 2005), UAS-eyg #2 BL26809 (Jang et al., 2003). UAS-Pax6 (5a) (Dominguez et al., 2004), UAS-lacZ (BL1777), 30A-gal4 (BL37534). Ato3.6BB-GFP (Tanaka-Matakatsu et al., 2014), Da-Gal4 (Wodarz et al., 1995), UAS-Da-Da (Tanaka-Matakatsu et al. 2014), dpp-lacZ BS3.0 (BL5528), *yw*, *hsFLP*; *AyGAL4*, *UAS-GFP*/TM6b, Tb.

### Histochemistry

Imaginal disc Immunohistochemistry and *in situ* hybridization were performed as previously described (Tanaka-Matakatsu and Du, 2008). Primary antibodies were used at following dilutions: rabbit α-GFP 1:1000 (GenScript), mouse α-GFP 1:500 (BD Bioscience), and rabbit α-Ey (Halder et al., 1998). Dye conjugated secondary antibodies were from Jackson ImmunoResearch and used at 1:500 dilution: goat α-mouse Cy2, goat α-rabbit-Cy2. DAPI used at 1:100 (5 µg/mL) for DNA staining. Images were taken using a Zeiss AxioImager microscope with ApoTome.

### Transgenic flies

The ey-PB cDNA (GH01157, DGRC) was subcloned into pUAST vector. Multiple transgenic lines were established using pTurbo helper plasmids under standard injection procedure (Tanaka-Matakatsu and Du, 2008), and examined all lines to confirm the consistency.

### Statistical analysis

Freshly eclosed adult flies were collected and mounted on double stick tape for imaging under Leica MZFLIII stereomicroscope at zoom 3.2. Fifty hinges were counted for each genotype. For Ato reporter activity assay, wing discs were imaged under Zeiss AxioImager using 10× objective. Twenty wing discs were counted for each genotype. Images were taken at the same image acquisition settings for GFP area determination. The total pixel number in the area surrounding the Ventral Radius region was obtained in Photoshop CS3. For cell growth analysis, wing discs were imaged at 10× objective and pixel numbers were counted for each genotype. Minimum 50 clones were counted. The counted data were analyzed and created Box-and-Whisker plots in Excel. Whisker limit was at 1.5 x IQR, and the values over- or under-the 1.5 x IQR plotted as outliers.

### GST-pull down assay

DH5α that carries the respective GST-fusion plasmid were grown to OD_600_ = 0.6 and were induced with 0.1 mmol/L IPTG for 2 h at 37°C. GST or GST fusion proteins were immobilized to Glutathione sepharose beads. Bound proteins were blocked with 1 mg/mL BSA in 1× PBS supplemented with 1 mmol/L DTT, 1 mmol/L PMSF and 1% Triton X-100 for 30 min at 4°C before binding reaction. *In vitro* transcribed/translated proteins were labeled with ^35^S-Met using TNT T7 coupled Reticulocyte lysate System (Promega). Binding reaction was performed in 0.1 mg/mL BSA in 1× PBS for 2 h at 4°C. Samples were washed with 1× PBS supplemented with 0.2% NP-40, 1 mmol/L DTT and 1 mmol/L PMSF for 10 min, 3 times. Samples were resolved by SDS-PAGE and autoradiographed using STORM 860 Phosphorimager (Molecular Dynamics).

### Electrophoretic mobility shift assay

EMSA was performed as described before (Tanaka-Matakatsu and Du, 2008). Embryo extract was prepared from O/N embryo collection. Embryos were homogenized in 2 volumes of lysis buffer (500 mmol/L NaCl, 1% Triton X-100, 50 mmol/L Tris pH 6.0, Protease Inhibitor Cocktail (Roche) and 10 mmol/L PMSF) and centrifuged for 15 min at 12 K rpm at 4°C. Three microliters of supernatant was used for ^32^P labelled-Ey2 probe binding. Samples were resolved on 4% native PAGE (37.5:1 Acrylamide/Bis solution, Bio-Rad) in 0.5× TBE at 200 V for 2 h, and autoradiographed using STORM 860 Phosphorimager. See Supplemental primer list for oligo sequences.

## ABBREVIATIONS

Ato, Atonal; Ey, Eyeless; HD, homeodomain; PD, paired domain; Pax6, Paired box 6.

## Electronic supplementary material

Below is the link to the electronic supplementary material.

Supplementary material 1 (PDF 1288 kb)

## References

[CR1] Benassayag C, Plaza S, Callaerts P, Clements J, Romeo Y, Gehring WJ, Cribbs DL (2003). Evidence for a direct functional antagonism of the selector genes proboscipedia and eyeless in Drosophila head development. Development.

[CR2] Bessa J, Gebelein B, Pichaud F, Casares F, Mann RS (2002). Combinatorial control of Drosophila eye development by eyeless, homothorax, and teashirt. Genes Dev.

[CR3] Brand AH, Perrimon N (1993). Targeted gene expression as a means of altering cell fates and generating dominant phenotypes. Development.

[CR4] Campbell S, Inamdar M, Rodrigues V, Raghavan V, Palazzolo M, Chovnick A (1992). The scalloped gene encodes a novel, evolutionarily conserved transcription factor required for sensory organ differentiation in Drosophila. Genes Dev.

[CR5] Carriere C, Plaza S, Martin P, Quatannens B, Bailly M, Stehelin D, Saule S (1993). Characterization of quail Pax-6 (Pax-QNR) proteins expressed in the neuroretina. Mol Cell Biol.

[CR6] Chadwick R, Jones B, Jack T, McGinnis W (1990). Ectopic expression from the *Deformed* gene triggers a dominant defect in *Drosophila* adult head development. Dev Biol.

[CR7] Chao JL, Tsai YC, Chiu SJ, Sun YH (2004). Localized Notch signal acts through eyg and upd to promote global growth in *Drosophila* eye. Development.

[CR8] Chow RL, Altmann CR, Lang RA, Hemmati-Brivanlou A (1999). Pax6 induces ectopic eyes in a vertebrate. Development.

[CR9] Clements J, Hens K, Merugu S, Dichtl B, de Couet HG, Callaerts P (2009). Mutational analysis of the eyeless gene and phenotypic rescue reveal that an intact Eyeless protein is necessary for normal eye and brain development in *Drosophila*. Dev Biol.

[CR10] Czerny T, Schaffner G, Busslinger M (1993). DNA sequence recognition by Pax proteins: bipartite structure of the paired domain and its binding site. Genes Dev.

[CR11] Dominguez M, Ferres-Marco D, Gutierrez-Avino FJ, Speicher SA, Beneyto M (2004). Growth and specification of the eye are controlled independently by Eyegone and Eyeless in *Drosophila melanogaster*. Nat Genet.

[CR12] Duman-Scheel M, Weng L, Xin S, Du W (2002). Hedgehog regulates cell growth and proliferation by inducing Cyclin D and Cyclin E. Nature.

[CR13] Epstein J, Cai J, Glaser T, Jepeal L, Maas R (1994). Identification of a Pax paired domain recognition sequence and evidence for DNA-dependent conformational changes. J Biol Chem.

[CR14] Epstein JA, Glaser T, Cai J, Jepeal L, Walton DS, Maas RL (1994). Two independent and interactive DNA-binding subdomains of the Pax6 paired domain are regulated by alternative splicing. Genes Dev.

[CR15] Farhy C, Elgart M, Shapira Z, Oron-Karni V, Yaron O, Menuchin Y, Rechavi G, Ashery-Padan R (2013). Pax6 is required for normal cell-cycle exit and the differentiation kinetics of retinal progenitor cells. PLoS One.

[CR16] Gehring WJ (1996). The master control gene for morphogenesis and evolution of the eye. Genes Cells.

[CR17] Gehring WJ, Affolter M, Burglin T (1994). Homeodomain proteins. Annu Rev Biochem.

[CR18] Gibson G, Schier A, LeMotte P, Gehring WJ (1990). The specificities of Sex combs reduced and Antennapedia are defined by a distinct portion of each protein that includes the homeodomain. Cell.

[CR19] Glaser T, Walton DS, Maas RL (1992). Genomic structure, evolutionary conservation and aniridia mutations in the human PAX6 gene. Nat Genet.

[CR20] Greenwood S, Struhl G (1999). Progression of the morphogenetic furrow in the Drosophila eye: the roles of Hedgehog, Decapentaplegic and the Raf pathway. Development.

[CR21] Halder G, Callaerts P, Gehring WJ (1995). Induction of ectopic eyes by targeted expression of the eyeless gene in Drosophila. Science.

[CR22] Halder G, Callaerts P, Flister S, Walldorf U, Kloter U, Gehring WJ (1998). Eyeless initiates the expression of both sine oculis and eyes absent during Drosophila compound eye development. Development.

[CR23] Hill RE, Favor J, Hogan BL, Ton CC, Saunders GF, Hanson IM, Prosser J, Jordan T, Hastie ND, van Heyningen V (1991). Mouse small eye results from mutations in a paired-like homeobox-containing gene. Nature.

[CR24] Jang CC, Chao JL, Jones N, Yao LC, Bessarab DA, Kuo YM, Jun S, Desplan C, Beckendorf SK, Sun YH (2003). Two Pax genes, eye gone and eyeless, act cooperatively in promoting *Drosophila* eye development. Development.

[CR25] Jarman AP, Groves AK (2013). The role of Atonal transcription factors in the development of mechanosensitive cells. Semin Cell Dev Biol.

[CR26] Jarman AP, Sun Y, Jan LY, Jan YN (1995). Role of the proneural gene, atonal, in formation of Drosophila chordotonal organs and photoreceptors. Development.

[CR27] Jaworski C, Sperbeck S, Graham C, Wistow G (1997). Alternative splicing of Pax6 in bovine eye and evolutionary conservation of intron sequences. Biochem Biophys Res Commun.

[CR28] Jiao R, Daube M, Duan H, Zou Y, Frei E, Noll M (2001). Headless flies generated by developmental pathway interference. Development.

[CR29] Jordan T, Hanson I, Zaletayev D, Hodgson S, Prosser J, Seawright A, Hastie N, van Heyningen V (1992). The human PAX6 gene is mutated in two patients with aniridia. Nat Genet.

[CR30] Jun S, Desplan C (1996). Cooperative interactions between paired domain and homeodomain. Development.

[CR31] Kim J, Lauderdale JD (2006). Analysis of Pax6 expression using a BAC transgene reveals the presence of a paired-less isoform of Pax6 in the eye and olfactory bulb. Dev Biol.

[CR32] Kiselev Y, Eriksen TE, Forsdahl S, Nguyen LH, Mikkola I (2012). 3T3 cell lines stably expressing Pax6 or Pax6(5a)–a new tool used for identification of common and isoform specific target genes. PLoS One.

[CR33] Kozmik Z, Czerny T, Busslinger M (1997). Alternatively spliced insertions in the paired domain restrict the DNA sequence specificity of Pax6 and Pax8. EMBO J.

[CR34] Li CG, Eccles MR (2012). PAX genes in cancer; friends or foes?. Front Genet.

[CR35] Mascarenhas JB, Young KP, Littlejohn EL, Yoo BK, Salgia R, Lang D (2009). PAX6 is expressed in pancreatic cancer and actively participates in cancer progression through activation of the MET tyrosine kinase receptor gene. J Biol Chem.

[CR36] Mikkola I, Bruun JA, Holm T, Johansen T (2001). Superactivation of Pax6-mediated transactivation from paired domain-binding sites by dna-independent recruitment of different homeodomain proteins. J Biol Chem.

[CR37] Mirzayans F, Pearce WG, MacDonald IM, Walter MA (1995). Mutation of the PAX6 gene in patients with autosomal dominant keratitis. Am J Hum Genet.

[CR38] Mishra R, Gorlov IP, Chao LY, Singh S, Saunders GF (2002). PAX6, paired domain influences sequence recognition by the homeodomain. J Biol Chem.

[CR39] Mismer D, Rubin GM (1989). Definition of cis-acting elements regulating expression of the *Drosophila* melanogaster ninaE opsin gene by oligonucleotide-directed mutagenesis. Genetics.

[CR40] Neufeld TP, de la Cruz AF, Johnston LA, Edgar BA (1998). Coordination of growth and cell division in the Drosophila wing. Cell.

[CR41] Nornes S, Clarkson M, Mikkola I, Pedersen M, Bardsley A, Martinez JP, Krauss S, Johansen T (1998). Zebrafish contains two pax6 genes involved in eye development. Mech Dev.

[CR42] Ostrin EJ, Li YM, Hoffman K, Liu J, Wang KQ, Zhang L, Mardon G, Chen R (2006). Genome-wide identification of direct targets of the Drosophila retinal determination protein Eyeless. Genome Res.

[CR43] Papatsenko D, Nazina A, Desplan C (2001). A conserved regulatory element present in all Drosophila rhodopsin genes mediates Pax6 functions and participates in the fine-tuning of cell-specific expression. Mech Dev.

[CR44] Plaza S, Langlois MC, Turque N, LeCornet S, Bailly M, Begue A, Quatannens B, Dozier C, Saule S (1997). The homeobox-containing Engrailed (En-1) product down-regulates the expression of Pax-6 through a DNA binding-independent mechanism. Cell Growth Differ.

[CR45] Plaza S, Prince F, Jaeger J, Kloter U, Flister S, Benassayag C, Cribbs D, Gehring WJ (2001). Molecular basis for the inhibition of Drosophila eye development by Antennapedia. EMBO J.

[CR46] Punzo C, Kurata S, Gehring WJ (2001). The eyeless homeodomain is dispensable for eye development in Drosophila. Gene Dev.

[CR47] Punzo C, Seimiya M, Flister S, Gehring WJ, Plaza S (2002). Differential interactions of eyeless and twin of eyeless with the sine oculis enhancer. Development.

[CR48] Quiring R, Walldorf U, Kloter U, Gehring WJ (1994). Homology of the eyeless gene of Drosophila to the Small eye gene in mice and Aniridia in humans. Science.

[CR49] Robson EJ, He SJ, Eccles MR (2006). A PANorama of PAX genes in cancer and development. Nat Rev Cancer.

[CR50] Sheng G, Thouvenot E, Schmucker D, Wilson DS, Desplan C (1997). Direct regulation of rhodopsin 1 by Pax-6/eyeless in *Drosophila*: evidence for a conserved function in photoreceptors. Genes Dev.

[CR51] Silver SJ, Rebay I (2005). Signaling circuitries in development: insights from the retinal determination gene network. Development.

[CR52] St-Onge L, Sosa-Pineda B, Chowdhury K, Mansouri A, Gruss P (1997). Pax6 is required for differentiation of glucagon-producing alpha-cells in mouse pancreas. Nature.

[CR53] Sukhanova MJ, Deb DK, Gordon GM, Matakatsu MT, Du W (2007). Proneural basic helix-loop-helix proteins and epidermal growth factor receptor signaling coordinately regulate cell type specification and cdk inhibitor expression during development. Mol Cell Biol.

[CR54] Tanaka-Matakatsu M, Du W (2008). Direct control of the proneural gene atonal by retinal determination factors during Drosophila eye development. Developmental Biology.

[CR55] Tanaka-Matakatsu M, Miller J, Borger D, Tang WJ, Du W (2014). Daughterless homodimer synergizes with Eyeless to induce Atonal expression and retinal neuron differentiation. Dev Biol.

[CR56] Treisman J, Harris E, Desplan C (1991). The paired box encodes a second DNA-binding domain in the paired homeo domain protein. Genes Dev.

[CR57] van Heyningen V, Williamson KA (2002). PAX6 in sensory development. Hum Mol Genet.

[CR58] Walcher T, Xie Q, Sun J, Irmler M, Beckers J, Ozturk T, Niessing D, Stoykova A, Cvekl A, Ninkovic J (2013). Functional dissection of the paired domain of Pax6 reveals molecular mechanisms of coordinating neurogenesis and proliferation. Development.

[CR59] Weasner BM, Weasner B, Deyoung SM, Michaels SD, Kumar JP (2009). Transcriptional activities of the Pax6 gene eyeless regulate tissue specificity of ectopic eye formation in Drosophila. Dev Biol.

[CR60] Wodarz A, Hinz U, Engelbert M, Knust E (1995). Expression of crumbs confers apical character on plasma membrane domains of ectodermal epithelia of Drosophila. Cell.

[CR61] Wolf LV, Yang Y, Wang J, Xie Q, Braunger B, Tamm ER, Zavadil J, Cvekl A (2009). Identification of pax6-dependent gene regulatory networks in the mouse lens. PLoS One.

[CR62] Xie Q, Yang Y, Huang J, Ninkovic J, Walcher T, Wolf L, Vitenzon A, Zheng D, Gotz M, Beebe DC (2013). Pax6 interactions with chromatin and identification of its novel direct target genes in lens and forebrain. PLoS One.

[CR63] Xin S, Weng L, Xu J, Du W (2002). The role of RBF in developmentally regulated cell proliferation in the eye disc and in Cyclin D/Cdk4 induced cellular growth. Development.

[CR64] Xu W, Rould MA, Jun S, Desplan C, Pabo CO (1995). Crystal structure of a paired domain-DNA complex at 2.5 A resolution reveals structural basis for Pax developmental mutations. Cell.

[CR65] Xu HE, Rould MA, Xu W, Epstein JA, Maas RL, Pabo CO (1999). Crystal structure of the human Pax6 paired domain-DNA complex reveals specific roles for the linker region and carboxy-terminal subdomain in DNA binding. Genes Dev.

[CR66] Yao JG, Sun YH (2005). Eyg and Ey Pax proteins act by distinct transcriptional mechanisms in Drosophila development. EMBO J.

[CR67] Yao LC, Liaw GJ, Pai CY, Sun YH (1999). A common mechanism for antenna-to-Leg transformation in Drosophila: suppression of homothorax transcription by four HOM-C genes. Dev Biol.

[CR68] Zhang TY, Ranade S, Cai CQ, Clouser C, Pignoni F (2006). Direct control of neurogenesis by selector factors in the fly eye: regulation of atonal by Ey and So. Development.

