# Supplementary material for: The homeodomain of Eyeless regulates cell growth and antagonizes the paired domain-dependent retinal differentiation function
Source: Protein Cell. 2014 Sep 20;6(1):68–78. doi: 10.1007/s13238-014-0101-9 (PMC4286722; doi:10.1007/s13238-014-0101-9)
Supplement: Supplementary file 1 — Supplementary material 1 (PDF 1288 kb) [file 13238_2014_101_MOESM1_ESM.pdf]

SUPPLEMENTARY FILE

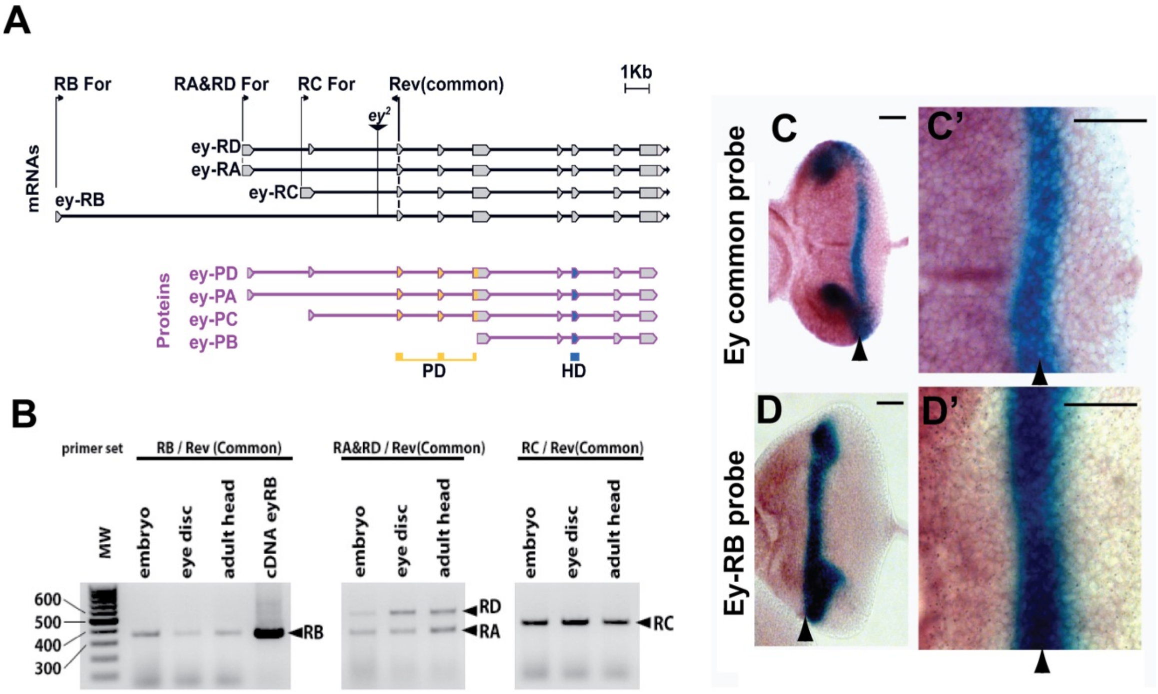

Supplemental Figure1. Ey HD type isoform express in the anterior domain in the developing eye disc. (A)Schematics of alternative spliced Ey. Exon (gray box) and intron (black line) structures are shown. Three forward primers used for RT-PCR are indicated with black forward oriented arrows. Common reverse primer position is indicated on reverse oriented arrow. Alternatively produced four Ey proteins are shown in below Ey-PA to Ey-PD in magenta color. Ey-PB protein contains only Homeodomain (highlighted with blue), other three alternatively produced Ey proteins contain both Paired Domain (highlighted with yellow) and Homeodomain. (B) RT-PCR analysis shows mRNA levels of ey alternative spliced products. Total RNA were isolated from O/N embryo collection, the 3rd instar eye discs, and adult heads were reversed transcription. All Flybase reported four Ey isoforms were detected in this analysis. (C and D) *in situ* hybridization with ey probe in the 3rd instar eye discs. Ey-RB (HD type isoform,

brown staining in D and D') expresses anterior to the MF (black arrows), this expression pattern is identical to the all other ey isoforms expression pattern (ey common, dark puple in C and C'). The morphogenetic furrow labelled with the expression of dpp-lacZ (blue). The scale bars are 50µm.

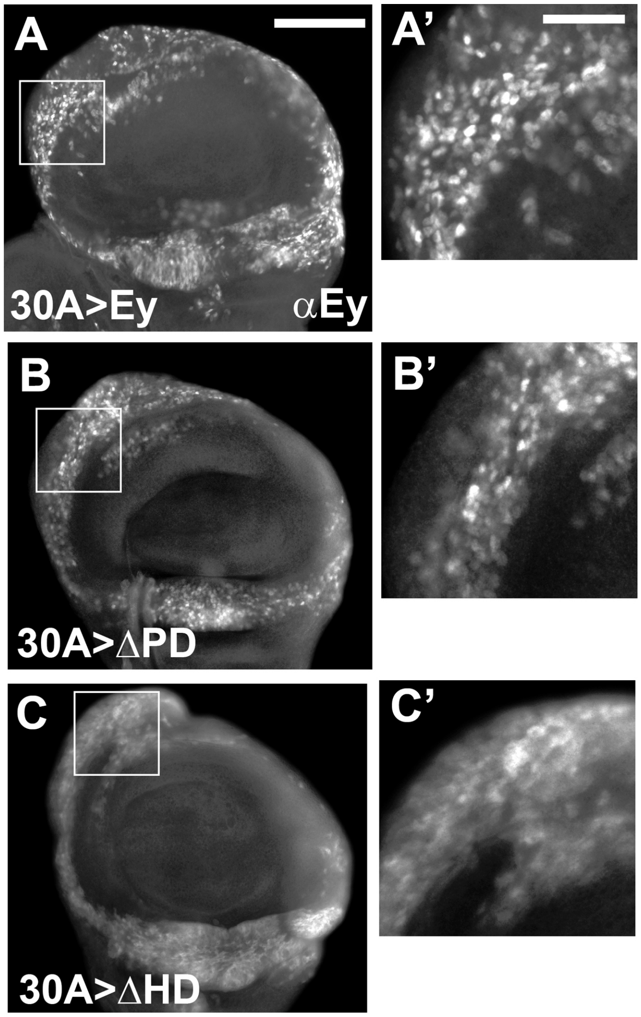

Supplemental Figure2. Expression levels of Ey deletion constructs. (A) 30A>Ey wing discs stained with α-Ey. (B and C) Expression of Ey deletion constructs ΔPD and ΔHD under 30A gal4 driver at identical condition to 30A>Ey. Images were taken at x20

magnification in (A-C). (A'-C') Photoshop enlarged images in boxed area in A-C are shown. The scale bars are 50μm in A-C, and 150μm in A'-C'.

SUPPLEMENTARY MATERIALS AND METHODS

RT-PCR

Total RNA was prepared from embryo using TRIZOL reagent followed by manufacturer's protocol. Reaction performed as described before (Tanaka-Matakatsu et al., 2009). The first exon of the Ey-RB mRNA used to generate in situ probe. The last 4 exon sequences were used for ey common in situ probe. Primer sequences are shown in the primer list.

PRIMER LIST

Experiment	Primer	Sequence
Ey RT-PCR	Ey-RA&RD Forward	5'- TTCGCACGGCGTGCGTTTGG-3'
	Ey-RB Forward	5'- TCCAATCGATACTACAAAATACC-3'
	Ey-RC Forward	5'- GAATTCCAAGTACAAACTGAC-3'
	Ey-common Reverse	5'- CGAGAATTTTGCTCACACATCC-3'
Ey-RB in situ probe	Ey-RB unique reverse	5'-ATTTATATTACGAATGG-3'<br>Used Ey-RB forward primer
EMSA	Ey2 (WT)Forward	5'- GATACAGTTTTCCAGCTCATTTGCTTGT AATTGGGCACCAA-3'
	Ey2(WT) Reverse	5'-TTGGTGCCCAATTACAAGCAAATGAGCT GGAAAACTGTATC-3'

construct	Primer name	Primer Sequence or origin of subcloning fragment
pUAST -Ey-PB	EyPB-XhoI Forward	5'- CTTCCT CGAGCAAAATGCAGACAGCC-3'
	EyPB-XbaI Reverse	5'- CGCCTAGTCTAGACTAGACCCACGGTG-3'

Ey/pET28b		Ey coding sequence from pUAST-Ey was subcloned into pET28b vector
PD/pET28c	EyPD HindIII Forward	5'-CGGCCGAAGCTTTCAAAATGTTTACATTGC-3'
	EyPD XhoI Reverse	5'-TCTAGGATCTCGAGCGTGCTTTGC-3'
HD/pET28b	EyHD HindIII Kozak Forward	5'-CGCCGCAAGCTTGCAAAAATGGAGGATGATC-3'
	EyHD XhoI Reverse	5'-AGTCATCTCGAGCTATGGTGTTCTTCGC-3'
ΔPD/pET28b	ΔPD Forward	5'-GCGCAAAAGGAGCAGCAAAGCACGGG-3'
	ΔPD Reverse	5'-CTGCTCCTTTTGCGCCAAAGGCCTTCC-3'
ΔHD/pET28b	ΔHD Forward	5'-AAGCTGCGAAACCAGCGAAGAACACC-3'
	ΔHD Reverse	5'-CGCTGGTTTCGCAGCTTCTTTCTTTTTAG-3'
ΔPDΔHD /pET28b		N-terminal ΔPD fragment was subcloned into ΔHD/pET 28b plasmid
EyN /pGEX4T2	Ey5' BglII Forward	5'- CGCAGATCTATGTTTACATTGCAACC-3'
	EyN BglII Reverse	5'- CGCAGATCTGGAATTTGGTGTTCTTC-3'
PD /pGEX4T2	PDβ-GST-BglII Forward	5'- CATGGCTAGATCTGGTCACAGTGGAGT AAATC-3'
	EyPD XhoI Reverse	5'- TCTAGGATCTCGAGCGTGCTTTGC-3'
N-PAI /pGEX4T2 #3		EcoRI fragment from N-term-PD/pGEX4T2
RED /pGEX4T2 #6		BamHI-XhoI fragment from PD/pGEX4T2
HD /pGEX4T2	EyHD-GST BglII Forward	5'-GCCAGAGATCTCAAATGGAGGATGATC-3'
	EyHD XhoI stop Reverse	5'-AGTCATCTCGAGCTATGGTGTTCTTCGC-3'
HD h1-2 /pGEX4T2 #12		EcoRI digest from HD/pGEX4T2 (120bp fragment) and subcloned into pGEX4T2 at EcoRI site
HD h3 /pGEX4T2 #20		EcoRI /XhoI digest from HD/pGEX4T2 and subcloned into pGEX4T2
